# Treatment burden and health literacy among patients referred to a vascular access clinic: a cross-sectional study

**DOI:** 10.1186/s12882-025-04594-2

**Published:** 2025-11-25

**Authors:** Ben Edgar, Catrin Jones, Laura Martin, Karen Stevenson, Peter C. Thomson, Patrick B. Mark, David B. Kingsmore

**Affiliations:** 1 School of Cardiovascular & Metabolic Health, University of Glasgow, Glasgow, UK; 2 School of Health & Wellbeing, University of Glasgow, Glasgow, UK; 3 Renal Surgery and Transplant Unit, Queen Elizabeth University Hospital, Glasgow, G51 4TF UK; 4 Department of Vascular Surgery, Queen Elizabeth University Hospital, Glasgow, UK

**Keywords:** Treatment burden, Health literacy, Dialysis access, Chronic kidney disease, Patient-centred care

## Abstract

**Background:**

Treatment burden, defined as ‘the work of being a patient’, can have implications on clinical outcomes and quality of life. Delivering minimally burdensome care requires recognition of the distribution of treatment burden in specific patient populations, and consideration of its key drivers whilst designing healthcare services.

**Methods:**

A prospective, cross-sectional study was performed to assess treatment burden among patients attending a regional vascular access surgery clinic over a 2-year period. Health literacy was synchronously measured to assess patients’ ability to process written information in the clinic.

**Results:**

A total of 563 patients were included (median age 64 years; 57% male), of whom 263 were receiving kidney replacement therapy (KRT). One in five patients (20%; 113/563) reported treatment burden levels indicative of being at risk of becoming overwhelmed by their care. Higher treatment burden was associated with dialysis dependence and greater socioeconomic deprivation. On multivariate analysis, poor health literacy was independently associated with unsustainable treatment burden (OR 3.78, *p* < 0.001).

**Conclusions:**

High treatment burden is prevalent among vascular access patients, particularly those with limited health literacy. Interventions that deliver patient-centred information in ways that do not depend on high literacy levels may help reduce treatment burden and support shared decision-making in this setting.

## Key learning points

What was known

What this study adds

Potential impact

High treatment burden is associated with reduced medication adherence and poorer health-related quality of life. However, the distribution and key drivers of treatment burden have not been descriptively analysed in patients referred for dialysis access surgery.

In our centre, 20% of patients referred for dialysis access surgery reported levels of treatment burden considered unsustainable. Poor health literacy was the strongest independent predictor of high burden, with medication management, administrative complexity, and financial strain identified as key contributing factors.

Implementing patient-centred communication strategies that do not rely on high health literacy may help reduce perceived treatment burden, enhance patient understanding of the healthcare journey, and support shared decision-making in this vulnerable cohort.

## Introduction

Chronic kidney disease (CKD) is a significant contributor to global morbidity and mortality, with a prevalence that continues to rise worldwide [1]. While early stages of CKD are often asymptomatic, progression to kidney failure is associated with an increasing symptom burden and a corresponding decline in health-related quality of life [2].

Beyond physical symptoms, patients with CKD face the ongoing challenges of self-management. These include coordinating appointments, adhering to complex medication regimens, understanding lifestyle and dietary recommendations, and navigating the financial demands of managing a chronic condition. Multimorbidity is common, and patients are frequently required to manage multiple treatment plans simultaneously, often requiring substantial time, cognitive effort, and emotional resilience. These demands collectively contribute to a patient’s treatment burden - the impact which the ‘work of being a patient’ has on their functioning and well-being [3].

Patients approaching kidney replacement therapy (KRT) initiation must contend with an even greater informational and logistical burden. They often face frequent appointments with nephrologists, vascular access and transplant surgeons, dialysis nurses, dietitians, and pharmacists. This can overwhelm patients with information, impairing their ability to process and remember what is discussed during appointments [4]. While written materials may supplement discussions, a proportion of patients may lack the health literacy required to fully understand and act upon such information.

Importantly, a patient’s perceived treatment burden is not solely a function of their clinical complexity but also of their capacity to manage the demands of their healthcare. This is a dynamic entity shaped by individual circumstances such as social support, socioeconomic status, and the resources available to a patient at any given time [5]. As a result, two patients with similar diagnoses and treatment regimens may report vastly different experiences of burden.

The aim of this study was to measure health literacy and perceived treatment burden in a cohort of patients referred to a haemodialysis vascular access surgery clinic, and to identify factors associated with unsustainable levels of treatment burden in this population.

## Methods

### Study design and population

A cross-sectional study was conducted to assess health literacy and perceived treatment burden among patients attending a regional vascular access surgery clinic. The study population included patients with pre-dialysis CKD and patients receiving KRT who were referred for new vascular access creation or evaluation of existing access issues.

This study was conducted at a tertiary referral centre providing renal transplant and vascular access services to the West of Scotland. As part of routine service improvement, clinical care questionnaires were completed by patients attending the clinic between 1st May 2023 and 31st May 2025, and the results retrospectively analysed following the data collection period. All patients attending the clinic during this period were eligible for inclusion; however, questionnaires were available only in English.

### Questionnaires

All patients attending the clinic were approached by a member of non-clinical administrative staff and asked to complete a Treatment Burden Questionnaire (TBQ) and a single-item health literacy screening questionnaire (SILS) prior to their appointment with a clinician.

#### Treatment Burden Questionnaire

The Treatment Burden Questionnaire is a validated patient-reported outcome measure (PROM) designed to assess the perceived burden associated with managing chronic conditions. It captures burden across several domains, including medication use, medical appointments and investigations, administrative and financial strain, and the impact of treatment on relationships and daily life. The TBQ consists of 15 items scored on a Likert-type scale ranging from 0 (“not a problem”) to 10 (“a big problem”), generating a total score out of 150. A total score of 59 or greater was used to define unsustainable treatment burden. This threshold corresponds to the “patient acceptable symptom state” (PASS) established in the original validation study, representing the score below which 75% of patients reported an acceptable level of treatment burden. Patients scoring above this PASS score were therefore considered at high risk of becoming overwhelmed by their medical care, and the cutoff of 59 has been adopted in this study to identify those experiencing unsustainable treatment burden [6].

Originally developed for patients with multimorbidity, the TBQ has since been validated in populations with chronic kidney disease (CKD) and kidney failure, demonstrating good construct validity, internal consistency, and test–retest reliability [7, 8]. 

#### Single Item Literacy Screener (SILS)

The SILS is a validated screening tool designed to identify individuals who may have difficulty understanding written health information. It is a condensed form of the Short Test of Functional Health Literacy in Adults (S-TOFHLA) and provides a rapid assessment of functional health literacy. The screener asks: *“How often do you need to have someone help you when you read instructions*,* pamphlets*,* or other written material from your doctor or pharmacy?”.*

Response options include: *Never*, *Rarely*, *Sometimes*, *Often*, and *Always*. Responses of *Sometimes*, *Often*, or *Always* have been associated with limited reading ability and poor health literacy [9].

### Data collection

Anonymised data collection was conducted following Caldicott Guardian approval. Patient demographics including age, sex, primary renal disease, frailty, medical comorbidities, and the number of prescribed medications were collected from a prospectively maintained electronic patient record.

Medical comorbidity was quantified using the Charlson Comorbidity Index (CCI) [10], a validated tool that classifies comorbid conditions associated with mortality risk. Comorbidity severity was categorised as mild (CCI 1–2), moderate (3–5), or severe (>5).

The number of prescribed medications reflects all repeat prescriptions in place at the time of questionnaire completion. Given the high prevalence of polypharmacy in the CKD population [11], categorical analyses in this study define ‘polypharmacy’ as a number of prescribed medications exceeding the cohort median.

Socioeconomic deprivation was assessed using the Scottish Index of Multiple Deprivation (SIMD), which assigns a relative deprivation score to nearly 7,000 data zones across Scotland [12]. The SIMD incorporates seven domains: income, employment, education, health, access to services, crime, and housing. These scores are relative to the overall Scottish population rather than the study cohort alone.

Travel times were estimated using the Google Maps Application Programming Interface (API). An anonymised list of participant postcodes was geocoded to generate approximate location coordinates. To protect confidentiality, coordinates were spatially masked using a random jittering technique. Driving times from each masked location to the hospital attended were then estimated by querying the API. This approach provides a reasonable approximation of travel burden while ensuring that no individually identifiable location data were used. Although less precise than address-level geocoding, this method was necessary to maintain participant anonymity [13, 14]. 

### Statistical analysis

Data were collated using Microsoft Excel (Version 16.53, © Microsoft, 2021), and statistical analyses conducted using RStudio (Version 2023.06.2 + 561, © Posit Software, PBC, 2022). The distribution of continuous variables was assessed using the Shapiro–Wilk test and means or medians calculated. Group comparisons were performed using the Wilcoxon rank-sum test for continuous variables and Pearson’s Chi-squared or Fisher’s exact test for categorical variables, as appropriate.

Partially completed forms were excluded from analyses of global treatment burden but were retained for item-level analysis. Responses marked ‘Not Applicable’ were scored as 0, following guidance published with the TBQ [15].

Skewness in questionnaire responses was quantified using the Fisher–Pearson coefficient, chosen for its sensitivity to outliers and its utility in characterising distributional asymmetry. This approach allowed exploration of variation in treatment burden beyond central tendency, identifying tasks that disproportionately affect a subset of patients. Items with higher positive skew indicate that while most patients report low burden, some experience very high burden, highlighting aspects of care that may require targeted support. To identify potential contributors to perceived treatment burden, skewness values for individual TBQ items were compared between participants with sustainable and unsustainable burden scores.

Independent predictors of unsustainable treatment burden were identified using multivariable logistic regression. Variables demonstrating statistical significance in univariate analyses were included in the final model.

## Results

A total of 563 questionnaires were completed over a two-year period. The study population included both incident haemodialysis patients (those with CKD stage 5 referred for consideration of vascular access) and prevalent KRT patients (those already established on dialysis or with a prior transplant, attending clinic for new or problematic access).

The cohort was 57% male, with a median age of 64 years, and included a range of primary renal diseases. Most participants had moderate to severe comorbidity. Socioeconomic data showed that over one-third of patients were from the most deprived 20% of the Scottish population (Table 1).

**Table 1:** Demographics of study cohort

	Overall *n* = 563	Incident *n* = 300	Prevalent *n* = 263
**Gender**, *n (%)*			
Male	322 (57%)	175 (58%)	147 (56%)
**Age**, *years*			
Median (IQR)	64 (51, 71)	65 (54, 72)	63 (50, 70)
Range	19–90	19–87	21–90
**Primary Renal Diagnosis**, *n (%)*			
Diabetes	165 (29%)	97 (32%)	68 (26%)
Glomerulonephritis	131 (23%)	60 (20%)	71 (27%)
Other^1^	175 (31%)	83 (28%)	92 (35%)
CKD of Unknown Origin	92 (16%)	60 (20%)	32 (12%)
**Charlson Comorbidity Index**, *n (%)*^**2**^			
Mild	20 (3.6%)	9 (3.0%)	11 (4.2%)
Moderate	210 (37%)	110 (37%)	100 (38%)
Severe	333 (59%)	181 (60%)	152 (58%)
**Socioeconomic Deprivation**, *n (%)*^**3**^			
Quintile 1 - Most Deprivation	190 (34%)	106 (35%)	84 (32%)
Quintile 2	119 (21%)	61 (20%)	58 (22%)
Quintile 3	80 (14%)	43 (14%)	37 (14%)
Quintile 4	96 (17%)	46 (15%)	50 (19%)
Quintile 5 - Least Deprivation	77 (14%)	43 (14%)	34 (13%)
**Kidney Replacement Therapy**, *n (%)*			
Hospital HD	208 (37%)	n/a	208 (79%)
Peritoneal Dialysis	5 (0.9%)	n/a	5 (1.9%)
Transplant	48 (8.5%)	n/a	48 (18%)
**KRT Time**, *years*			
Median (IQR)	2 (1, 8)	0 (0, 0)	2 (1, 8)

^1^Alport’s Syndrome, Amyloidosis, Autosomal Dominant Polycystic Kidney Disease, Congenital abnormality, Cortical necrosis, Drug-Induced, Hypertensive nephropathy, Ischaemic nephropathy, Myeloma, Obstructive nephropathy

^2^ Mild = < 3, Moderate = 3–5, Severe = > 5

^3^ Scottish Index of Multiple Deprivation

### Health literacy

**Fig. 1:**
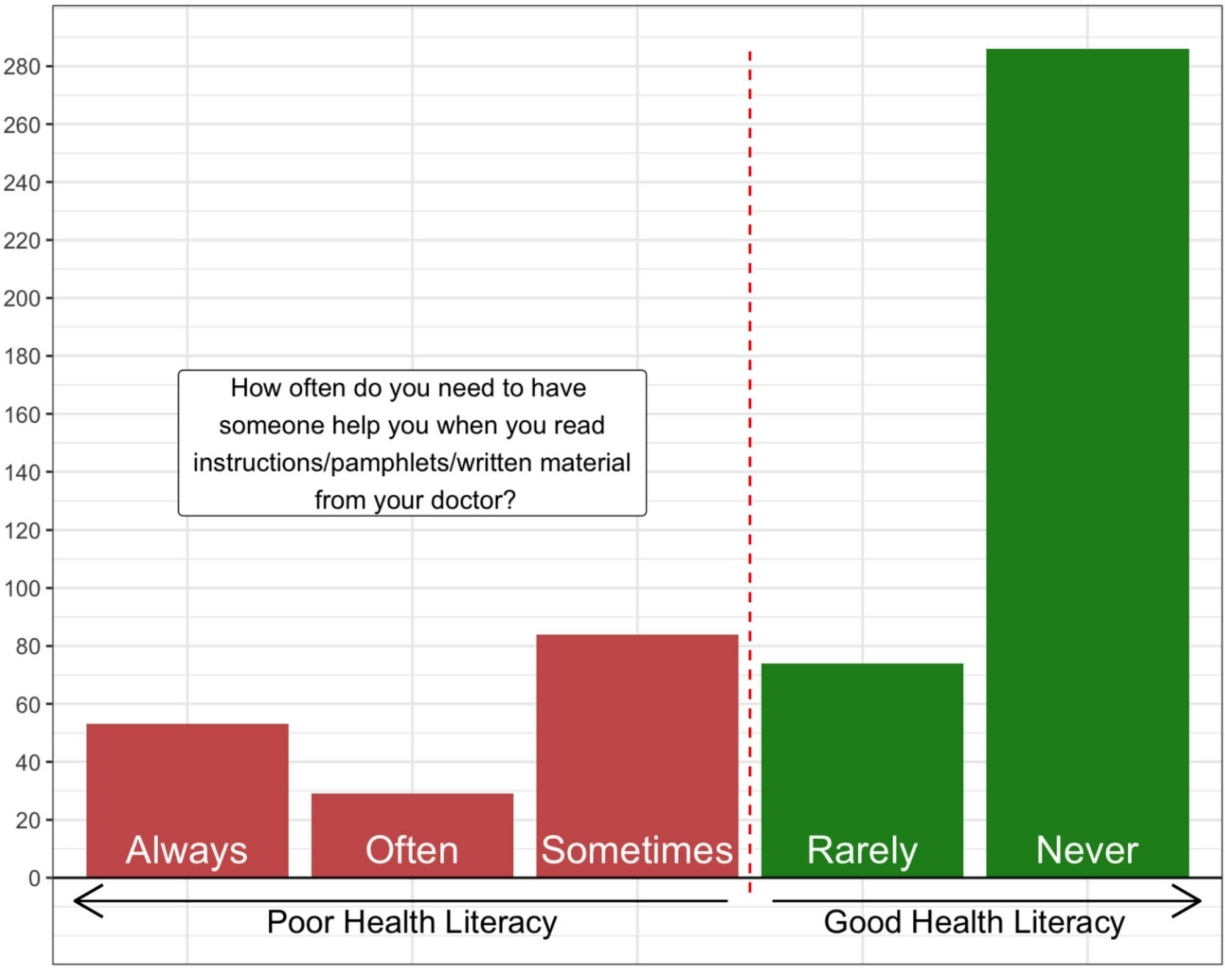
Single item literacy screening questionnaire scores

A total of 525 patients completed the Single Item Literacy Screener (SILS). The majority (68%) reported a good level of health literacy, defined as never or rarely requiring help to read written information provided by healthcare professionals (Fig. 1).

Patients with lower self-reported health literacy were significantly more likely to experience unsustainable treatment burden (35% vs. 12%, *p* < 0.001). This group was also more likely to live in areas of socioeconomic deprivation and to have a higher burden of medical comorbidity (Table 2).

**Table 2:** Comparing demographics and treatment burden based on health literacy

	Good health literacy^1^ *n* = 359	Poor health literacy^1^ *n* = 166	*p*-value^2^
**Age***(Median*,* IQR)*	65 (52, 72)	64 (53, 70)	> 0.9
**Gender**			0.8
Female	156 (43%)	70 (42%)	
Male	203 (57%)	96 (58%)	
**Charlson Comorbidity Index**			**0.004**
Mild	14 (3.9%)	3 (1.8%)	
Moderate	148 (41%)	47 (28%)	
Severe	197 (55%)	116 (70%)	
**Socioeconomic Deprivation**			**0.002**
1 - Most Deprived	107 (30%)	66 (40%)	
2	68 (19%)	44 (27%)	
3	54 (15%)	24 (14%)	
4	71 (20%)	20 (12%)	
5 - Least Deprived	59 (16%)	12 (7.2%)	
**Treatment Burden**			**< 0.001**
Sustainable	315 (88%)	108 (65%)	
Unsustainable	44 (12%)	58 (35%)	
**TBQ Completion**			**0.021**
Complete	305 (85%)	153 (92%)	
Partially complete	54 (15%)	13 (7.8%)	

^1^Single Item Literacy Screening: Good Literacy = “Never”, “Rarely”. Poor Literacy = “Always”, “Often”, “Sometimes”

^2^Wilcoxon rank sum test; Pearson’s Chi-squared test; Fisher’s exact test

Importantly, questionnaire completion rates were high in both groups (92% vs. 85%), suggesting that lower health literacy did not impair patients’ engagement with the assessment tools.

### Treatment burden

563 patients completed treatment burden questionnaires, however 72 were only partially completed.

The median total TBQ score was 27 (IQR 11–52; range 0–138), with 20% of respondents (113/563) scoring above 59—indicative of an unsustainable level of treatment burden.

**Fig. 2:**
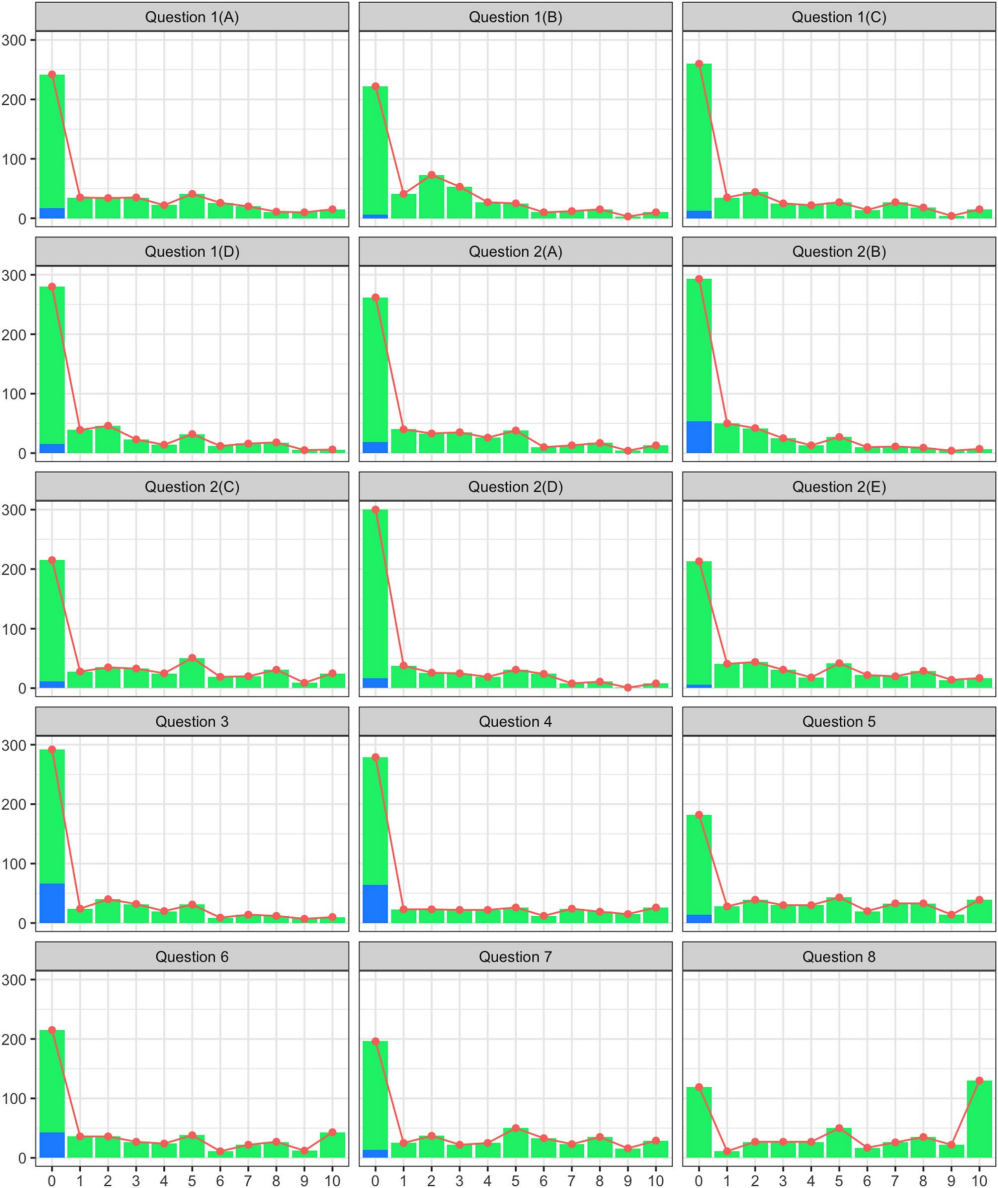
Answer distribution in whole study cohort. Key: Green = Answered. Blue = ‘Not Applicable’

**Fig. 3:**
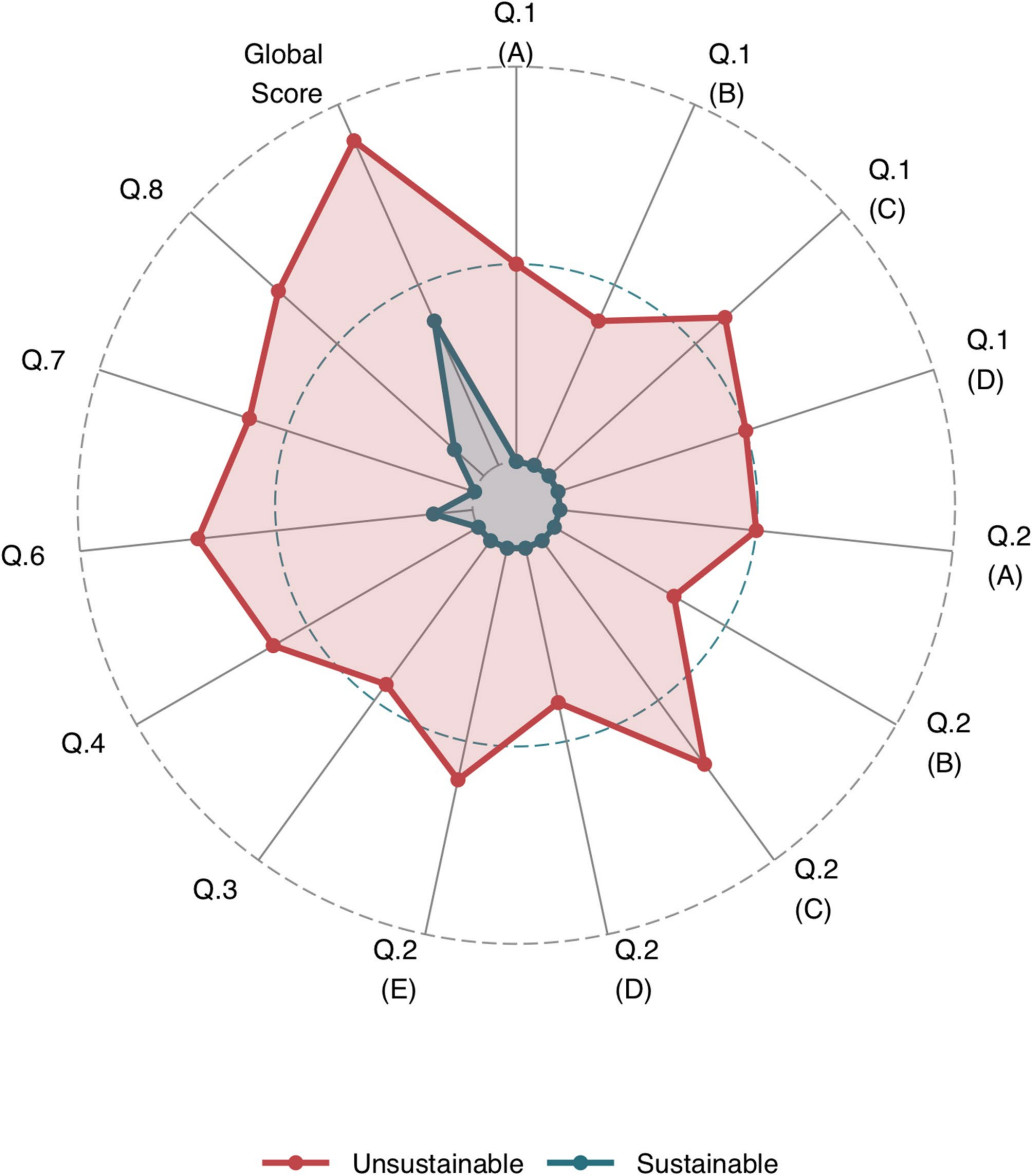
Median question responses for those with sustainable and unsustainable treatment burden scores. Key: Median scores from questionnaire responses per item. Red = patients with global scores above the threshold of sustainability, green = patients with scores below the threshold. Answers of ‘not applicable’ treated as zero

Median scores for individual items are presented in Table 3. Some questions, particularly those related to self-monitoring and administrative or financial burden, were more frequently marked as non-applicable (Fig. 2). Patients with treatment burden scores above the sustainability threshold consistently reported higher median item scores across nearly all questionnaire domains, indicating greater perceived burden compared to those below the threshold (Fig. 3). The item most commonly associated with high perceived burden concerned the constant reminder of illness due to ongoing healthcare needs.

**Table 3:** Median scores and perceived applicability for individual questions in the TBQ

	Question	Median score	Interquartile range	Proportion of ‘Not applicable’
**1.**	**How would you rate the problems related to:**			
*(A)*	The **taste**,** shape or size** of your tablets and/or **the annoyances** caused by your injections?	1	4.5	3.4%
*(B)*	The **number of times** you should take your medication daily?	1	3	1.2%
*(C)*	The **efforts you make not to forget to take your medications**?	0	4	2.6%
*(D)*	The **necessary precautions when taking your medication**?	0	3	3.1%
**2.**	**Regarding your medical follow-up**,** how would you rate the problems related to**:			
*(A)*	**Lab tests and other exams** (e.g. blood tests, radiology): frequency, time spent and associated nuisances or inconveniences?	0	3	3.8%
*(B)*	**Self-monitoring** (e.g. blood pressure, blood sugar): frequency, time spence and associated inconveniences?	0	2	**11%**
*€*	**Doctor visits and other appointments**: frequency and time spent for these visits and difficulties finding healthcare providers?	2	5	2.2%
*(D)*	The difficulties you could have in your **relationship with healthcare providers** (e.g. feeling not listened to or not taken seriously)?	0	3	3.5%
*€*	**Arranging medical appointments** (doctors visits, lab tests) and **reorganising your schedule** around these appointments?	1	5	1.2%
	**How would you rate**:			
**3**	The **administrative burden** related to healthcare (e.g. all you have to do for hospitalisations, reimbursements and/or obtaining social services)?	0	3	**13.4%**
**4**	The **financial burden** associated with your healthcare (e.g. out of pocket expenses or expenses not covered by insurance)?	0	4	**13%**
**5**	The burden related to **dietary changes** (e.g. avoiding certain foods or alcohol, having to quit smoking)?	2	6	2.8%
**6**	The burden related to **doctors’ recommendations to practice physical activity** (e.g. walking, swimming, jogging)?	1	5	8.7%
**7**	How does you healthcare impact **your relationships with others** (e.g. needing assistance in everyday life, being ashamed to take your medication)?	2	6	2.6%
**8**	“The need for medical healthcare on a regular basis **reminds me of my health problems**”	5	9	0%

**Fig. 4:**
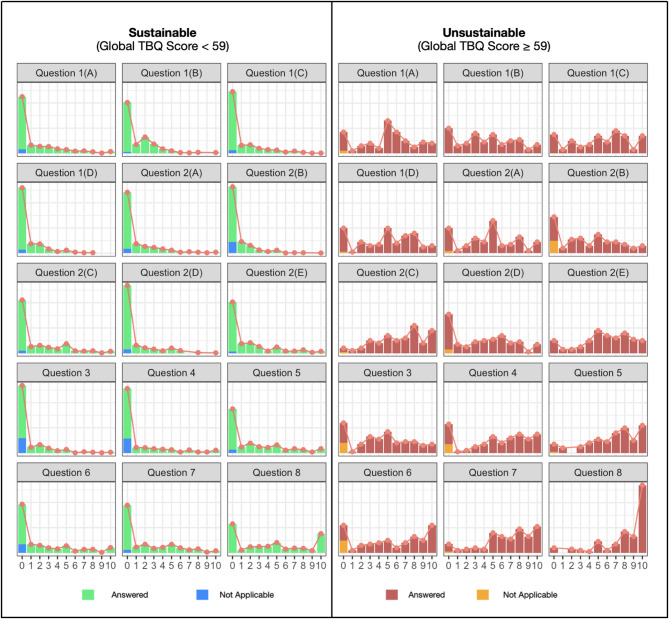
Distribution of answers in sustainable vs. unsustainable cohorts

To identify key drivers of treatment burden, skewness across item responses was analysed to capture differences not evident through central tendency measures. Items with the greatest absolute skewness differences between sustainable and unsustainable TBQ groups were considered the most influential. Larger positive skew indicates tasks experienced as highly burdensome by a minority of patients, even if mean scores are moderate, providing a practical guide for clinicians to prioritise areas for targeted support. The largest disparities were observed for medication-related challenges, including tablet characteristics, dosing frequency, and the effort required to avoid missed doses - highlighting these as primary contributors to treatment burden. Administrative and financial burdens also showed marked skew differences, underscoring the impact of organisational complexity and economic pressures on individuals’ treatment burden. In contrast, routine healthcare tests such as lab tests were somewhat less discriminatory. Overall, these findings suggest that medication management, administrative complexity, and financial strain are key drivers of unsustainable treatment burden in this study cohort (Fig. 4; Table 4).

**Table 4:** Analysis of skew in responses to questionnaires categorised by global TBQ score

Question	Skew		Difference in skew
	Sustainable *N* = 450	Unsustainable *N* = 113	
The **necessary precautions when taking your medication**?	2.19	-0.25	2.44
**Self-monitoring** (e.g. blood pressure, blood sugar): frequency, time spence and associated inconveniences?	2.50	0.18	2.32
The **financial burden** associated with your healthcare (e.g. out of pocket expenses or expenses not covered by insurance)?	1.72	-0.54	2.26
The **administrative burden** related to healthcare (e.g. all you have to do for hospitalisations, reimbursements and/or obtaining social services)?	2.20	0.007	2.19
The **efforts you make not to forget to take your medications**?	1.91	-0.27	2.18
**Lab tests and other exams** (e.g. blood tests, radiology): frequency, time spent and associated nuisances or inconveniences?	2.07	0.007	2.064
**Arranging medical appointments** (doctors visits, lab tests) and **reorganising your schedule** around these appointments?	1.55	-0.50	2.05
“The need for medical healthcare on a regular basis **reminds me of my health problems**”	0.26	-1.65	1.91
The **taste**,** shape or size** of your tablets and/or **the annoyances** caused by your injections?	1.68	-0.21	1.90
The difficulties you could have in your **relationship with healthcare providers** (e.g. feeling not listened to or not taken seriously)?	2.05	0.20	1.85
The burden related to **dietary changes** (e.g. avoiding certain foods or alcohol, having to quit smoking)?	1.02	-0.79	1.82
**Doctor visits and other appointments**: frequency and time spent for these visits and difficulties finding healthcare providers?	1.34	-0.46	1.81
How does your healthcare impact **your relationships with others** (e.g. needing assistance in everyday life, being ashamed to take your medication)?	0.99	-0.80	1.80
The **number of times** you should take your medication daily?	1.86	0.17	1.69
The burden related to **doctors’ recommendations to practice physical activity** (e.g. walking, swimming, jogging)?	1.14	-0.41	1.55

^1^Fisher’s g_1_: Positive values represent rightward skew, meaning most answers trend toward lower scores (e.g., 0). Negative values represent leftward skew, meaning most answers trend toward higher scores (e.g., 10)

Questions ordered by absolute difference in skew to identify key drivers of treatment burden in the unsustainable cohort

TBQ scores were significantly higher among prevalent kidney replacement therapy (KRT) patients compared to incident patients (median 29 vs. 25, *p* = 0.03), and unsustainable treatment burden was more common in this group (24% vs. 17%, *p* = 0.052). The largest absolute differences in response skew were observed for items related to reorganising life around appointments, attending healthcare visits, and medication characteristics. Other items showing notable differences included treatment complexity, administrative demands, and financial burden. These domains were all more burdensome in the prevalent group, suggesting that the logistical and systemic demands of care may intensify as patients transition onto kidney replacement therapy.

In univariable logistic regression analysis, several factors were significantly associated with unsustainable treatment burden. Younger age (OR 0.98, 95% CI 0.97–0.99, *p* = 0.006), polypharmacy (OR 1.58, 95% CI 1.03–2.43, *p* = 0.037), prevalent KRT (OR 1.57, 95% CI 1.03–2.40, *p* = 0.037), low health literacy (OR 3.73, 95% CI 2.37–5.94, *p* < 0.001), and greater socioeconomic deprivation (OR 1.38 per quintile, 95% CI 1.17–1.63, *p* < 0.001) were associated with higher odds of reporting unsustainable treatment burden.

In the multivariable model, low health literacy (adjusted OR 3.78, 95% CI 2.33–6.22, *p* < 0.001) and greater socioeconomic deprivation (adjusted OR 1.21, 95% CI 1.02–1.46, *p* = 0.034) remained independently associated with unsustainable treatment burden. Age, sex, prevalent KRT, primary renal diagnosis, Charlson comorbidity index, Clinical Frailty Score, residential distance to hospital, and driving time to appointments were not significantly associated with unsustainable treatment burden in the adjusted or unadjusted analyses (Table 5).

**Table 5:** Multivariable analysis: factors associated with unsustainable treatment burden

Characteristic	Measure	Sustainable burden	Unsustainable burden	OR (univariable)	OR (multivariable)
Health Literacy ^1^	Good n (%)	263 (85.9)	43 (14.1)	-	-
	Poor n (%)	95 (62.1)	58 (37.9)	3.73 (2.37–5.94, *p* < 0.001)	**3.78** **(2.33–6.22**, ***p*** < **0.001)**
Socioeconomic Deprivation (SIMD)^2^	Mean (SD)	2.7 (1.4)	2.1 (1.3)	1.38 (1.17–1.63, *p* < 0.001)	**1.21** **(1.02–1.46**, ***p*** = **0.034)**
Polypharmacy	Less than median	195 (81.2)	45 (18.8)	-	-
	More than median	164 (72.9)	61 (27.1)	1.57 (1.03–2.40, *p* = 0.037)	1.65 (0.98–2.80, *p* = 0.058)
Age	Mean (SD)	60.9 (13.7)	56.6 (15.3)	0.98 (0.97–0.99, *p* = 0.006)	0.99 (0.97-1.00, *p* = 0.109)
KRT Status	CKD V *n (%)*	215 (80.8)	51 (19.2)	-	-
	KRT *n (%)*	164 (72.9)	61 (27.1)	1.57 (1.03–2.40, *p* = 0.037)	1.31 (0.76–2.24, *p* = 0.332)
Sex				*p* = 0.11	Not included in model
Clinical Frailty Score				*P* = 0.6	Not included in model
Primary Renal Diagnosis				*p* = 0.11	Not included in model
Comorbidity				*p* = 0.6	Not included in model
Distance to hospital				*p* = 0.6	Not included in model
Driving time for appointment				*p* = 0.8	Not included in model

^1^ Single-Item Literacy Screener. Good = “Never” or “Rarely”. Poor = “Always”, “Often” or “Sometimes”

^2^per Quintile increase in deprivation. ^3^More than the median number of medications of the study cohort

## Discussion

An individual’s perception of treatment burden reflects not only the complexity of their healthcare demands but also their capacity to manage them. It is a dynamic construct, shaped by the evolving nature of their illness, personal circumstances, and the resources at their disposal. Its importance is increasingly recognised, with national multimorbidity guidelines advocating for an approach to care which includes ‘*improving quality of life by reducing treatment burden’* [16]. In our study, one in five patients attending the vascular access clinic reported a level of treatment burden deemed unsustainable - suggesting a perceived inability to maintain their current level of engagement with care over the long term.

This finding is consistent with previous studies in CKD and multimorbidity, in which high treatment burden has been associated with reduced adherence to medications and lower health-related quality of life [7, 17]. Importantly, the burden reported by patients in our cohort was not evenly distributed. Patients already established on renal replacement therapy were more likely to experience unsustainable burden than those approaching dialysis initiation, suggesting that perceived burden may increase as patients progress along the kidney care pathway. However, it should be noted that prevalent patients attending the vascular access clinic are not necessarily representative of the wider stable dialysis population. Many were referred because of access-related complications, the need for new or revised access, or changes in renal replacement modality. These circumstances may partly explain the higher levels of perceived burden observed among this group.

Our analysis of item-level skewness provides additional insight into the specific challenges contributing most to perceived burden in this cohort. Items with larger positive skew indicate that while most patients report low burden, some experience very high burden, highlighting areas where patients may struggle most. This approach helps clinicians and service designers identify specific aspects of care that may warrant targeted support, even when overall mean burden appears moderate. Medication-related tasks such as dealing with complex regimens, frequent dosing, and avoiding missed doses emerged as key contributors to unsustainable burden. These findings mirror previous literature highlighting polypharmacy as a major stressor in patients with advanced CKD [11]. In addition, the organisational complexity of care, including scheduling appointments and navigating administrative tasks were prominent drivers of treatment burden. In contrast, routine elements such as laboratory testing were less discriminative between those with sustainable and unsustainable levels of burden.

One of the most striking findings in our study was the strong association between low health literacy and unsustainable treatment burden. Patients who reported needing help to understand written health information were nearly four times more likely to experience high burden, even after adjusting for comorbidity, frailty, and socioeconomic status. This supports existing evidence linking limited health literacy to increased treatment burden in patients with cardiovascular disease [18–20], and may reflect a reduced capacity to comprehend, retain, and apply medical advice, ultimately complicating the management of chronic conditions. Encouragingly, completion rates for both health literacy and treatment burden assessments were high across the cohort, indicating that well-designed patient-reported outcome measures (PROMs) can still be accessible to patients with varying levels of literacy.

Socioeconomic deprivation was also independently associated with unsustainable burden. Patients from more deprived areas were significantly more likely to report high treatment burden, highlighting the role of patients’ social circumstances in shaping their capacity to manage the demands of healthcare. While clinical care may be similar across socioeconomic strata, the capacity to absorb demands such as transport costs, unpaid time off work, or caregiving responsibilities may differ substantially. Indeed, in this study patients from more deprived areas reported significantly higher scores on items relating to administrative (TBQ3) and financial (TBQ4) burden, suggesting these are key drivers of inequity in perceived treatment burden (Table 6).

**Table 6:** Comparison of TBQ item responses by level of socioeconomic deprivation

	Most deprivation^1^ *N* = 309		Least deprivation^1^ *N* = 173			
Question	Median (IQR)	Skew^2^	Median (IQR)	Skew^2^	X^2^	*p*-value
Q.1A	0.0 (0–5)	1.01	1 (0–4)	1.18	14.98	0.1328
Q.1B	2.0 (0–4)	1.20	1 (0–3)	1.27	17.25	0.0691
Q.1C	0.0 (0–4)	1.12	0 (0–3)	1.28	15.41	0.1178
Q.1D	0.0 (0–3)	1.38	0 (0–2)	1.76	10.99	0.3585
Q.2A	0.0 (0–4)	1.27	0 (0–3)	1.40	8.87	0.5443
Q.2B	0.0 (0–2)	1.71	0 (0–1)	1.85	17.48	0.0643
Q.2C	1.0 (0–5)	0.79	1 (0–5)	0.85	7.05	0.7205
Q.2D	0.0 (0–3)	1.56	0 (0–2)	1.69	13.44	0.1999
Q.2E	2.0 (0–5)	0.86	1 (0–4)	1.16	11.03	0.3555
Q.3	0.0 (0–3)	1.38	0 (0–2)	2.30	20.65	**0.0237**
Q.4	0.0 (0–5)	1.06	0 (0–2)	1.71	21.37	**0.0187**
Q.5	2.0 (0–7)	0.53	2 (0–5)	0.77	15.55	0.1133
Q.6	1.5 (0–6)	0.77	1 (0–5)	1.09	13.83	0.1810
Q.7	2.0 (0–6)	0.61	2 (0–5)	0.65	16.48	0.0868
Q.8	5.0 (2–10)	-0.18	5 (0–9)	0.10	10.51	0.3965

^1^Most Deprivation = SIMD Quintiles 1 & 2. Least Deprivation = SIMD Quintiles 4 & 5. ^2^Fishers g_1_

Vascular access remains a complex and potentially overwhelming topic for patients with CKD, particularly as the need for dialysis approaches. The increasing number of access options -including arteriovenous fistulas, tunnelled catheters, early-cannulation grafts, and novel techniques such as percutaneous AVF - can present a significant volume of information that may complicate shared decision-making, despite the recognised impact of initial access choice on long-term outcomes and patient experience [21, 22].

Effective communication strategies are therefore essential. Traditional written materials may be inadequate for patients with limited literacy and often fail to reflect the individualised and dynamic nature of vascular access planning. Digital platforms that incorporate illustrations and personalised content offer a promising means of improving patient understanding and supporting informed decision-making [23]. However, these tools must be implemented with care. Shifting information delivery to digital platforms may increase perceived burden for some individuals, particularly those with limited digital access or confidence. Ensuring that such solutions are accessible, inclusive, and complementary to in-person consultation will be key to their success [24–26].

This study has several strengths. It includes a large, real-world cohort of patients with advanced kidney disease, spanning both pre-dialysis and established KRT populations. The use of validated instruments—the Treatment Burden Questionnaire and Single Item Literacy Screener—adds methodological rigour, while the analysis of item-level skewness provides unique insight into variation within questionnaire domains. High response rates across literacy groups also support the feasibility of routine assessment of patient-reported outcome measures in clinical settings.

However, there are limitations. The cross-sectional design precludes causal inference, meaning that observed associations between patient factors and treatment burden cannot be interpreted as causal. In addition, this design does not capture changes in perceived burden over time. The study was conducted at a single tertiary centre in Scotland, which may limit generalisability to other settings. Moreover, the study was conducted within a universal healthcare system, where direct treatment costs are largely covered. In countries with fragmented or insurance-based systems, financial and administrative burdens may represent a greater component of overall treatment burden, and the distribution of unsustainable burden may therefore differ. As this study was conducted in a vascular access clinic serving predominantly haemodialysis patients, the findings primarily reflect the experiences of this group. Only a small number of participants were receiving peritoneal dialysis, and these individuals may not be representative of the broader PD population. The TBQ, although validated in CKD populations and showing excellent internal consistency in our analysis, was originally developed for multimorbidity more broadly and may not fully capture dialysis-specific aspects of treatment burden. A proportion of patients marked certain items as not applicable, and the TBQ lacks questions directly addressing aspects of renal replacement therapy. Similarly, the SILS, while validated and practical, assesses only a narrow domain of health literacy, specifically patients’ ability to understand written health information. It does not capture other relevant domains, including numeracy, digital literacy, or communication skills, which may also influence treatment burden in CKD. Finally, travel time estimates—though privacy-preserving—may not reflect real-world difficulties for patients dependent on public transport or carers.

An important consideration is that this study represents a retrospective analysis of routinely collected patient-reported outcome measures, including the SILS and TBQ, which are part of a quality improvement program aimed at optimizing service design and delivering minimally burdensome care. The SILS was not selected a priori for a detailed investigation of health literacy as a predictor of treatment burden; rather, low literacy emerged as an associated factor in this retrospective analysis. While the association observed is consistent with existing literature linking limited health literacy to difficulties with medication management, self-monitoring, and navigating complex care, it should be interpreted cautiously. Confirmation of this relationship would require a prospective study, ideally using a more comprehensive assessment of health literacy and potentially incorporating qualitative methods to explore how literacy influences perceived treatment burden. These approaches would provide a richer understanding of the mechanisms linking literacy to burden and inform more targeted interventions.

These findings have several practical implications. Both treatment burden and health literacy are measurable and potentially modifiable factors that can help identify patients at increased risk of disengagement or non-adherence. However the dynamic and context-dependent nature of treatment burden precludes its generalisation across patients with different conditions or in different countries [27]. Therefore, designing and delivering a minimally burdensome service requires first understanding how treatment burden is distributed within the specific patient population and identifying any common drivers contributing to it. Routinely assessing patient-reported outcome measures (PROMs) allows services to evaluate and adapt care—minimising unnecessary complexity and ensuring treatment pathways remain manageable for both individual patients and the broader population.

Beyond identifying patients at risk, addressing treatment burden in advanced CKD is likely to require a multifaceted approach that extends beyond improving health literacy alone. Interventions that combine care coordination, social work input, and health coaching to enhance patient activation and self-management have shown promise in reducing perceived burden and improving engagement in other chronic disease contexts. Such approaches recognise that treatment burden arises not only from the demands of care itself but also from the mismatch between these demands and the resources available to patients. While evaluation of these strategies was beyond the scope of the present study, our findings highlight the importance of identifying patients at risk of unsustainable burden as a first step towards designing and targeting supportive interventions that are responsive to their individual capacities and circumstances.

This study demonstrates that 20% of patients attending our vascular access surgery clinic perceive unsustainable levels of treatment burden, with poor health literacy emerging as an independent risk factor. Future longitudinal studies are needed to examine how treatment burden evolves over time and to identify which interventions are most effective in mitigating it, as well as to determine whether addressing specific burden domains can improve engagement, outcomes, and quality of life. Given the multifactorial and dynamic nature of treatment burden, such research will require adequately powered cohorts and careful adjustment for confounding factors but could provide valuable evidence to guide the design of sustainable, patient-centred kidney care pathways. Finally, alternative approaches to information delivery for patients with advanced CKD should be explored as potential strategies to reduce perceived burden and support more effective self-management.

## Data Availability

The data underlying this article will be shared on reasonable request to the corresponding author.

## Abbreviations

CCI: Charlson Comorbidity Index
CKD: Chronic Kidney Disease
KRT: Kidney Replacement Therapy
PROM: Patient–reported Outcome Measure
SILS: Single Item Literacy Screener
SIMD: Scottish Index of Multiple Deprivation
TBQ: Treatment Burden Questionnaire
